# A Physiologically Based Pharmacokinetic Model Relates the Subcutaneous Bioavailability of Monoclonal Antibodies to the Saturation of FcRn-Mediated Recycling in Injection-Site-Draining Lymph Nodes

**DOI:** 10.3390/antib13030070

**Published:** 2024-08-15

**Authors:** Felix Stader, Cong Liu, Abdallah Derbalah, Hiroshi Momiji, Xian Pan, Iain Gardner, Masoud Jamei, Armin Sepp

**Affiliations:** Simcyp Division, Certara UK Ltd., Level 2 Acero, 1 Concourse Way, Sheffield S1 2BJ, UKxian.pan@certara.com (X.P.); iain.gardner@certara.com (I.G.); armin.sepp@certara.com (A.S.)

**Keywords:** monoclonal antibodies, subcutaneous dosing, physiologically based pharmacokinetic modeling, antigen-presenting cells, neonatal Fc receptor (FcRn)

## Abstract

The bioavailability of a monoclonal antibody (mAb) or another therapeutic protein after subcutaneous (SC) dosing is challenging to predict from first principles, even if the impact of injection site physiology and drug properties on mAb bioavailability is generally understood. We used a physiologically based pharmacokinetic model to predict pre-systemic clearance after SC administration mechanistically by incorporating the FcRn salvage pathway in antigen-presenting cells (APCs) in peripheral lymph nodes, draining the injection site. Clinically observed data of the removal rate of IgG from the arm as well as its plasma concentration after SC dosing were mostly predicted within the 95% confidence interval. The bioavailability of IgG was predicted to be 70%, which mechanistically relates to macropinocytosis in the draining lymph nodes and transient local dose-dependent partial saturation of the FcRn receptor in the APCs, resulting in higher catabolism and consequently less drug reaching the systemic circulation. The predicted free FcRn concentration was reduced to 40–45%, reaching the minimum 1–2 days after the SC administration of IgG, and returned to baseline after 8–12 days, depending on the site of injection. The model predicted the uptake into APCs, the binding affinity to FcRn, and the dose to be important factors impacting the bioavailability of a mAb.

## 1. Introduction

Subcutaneous (SC) administration is increasingly used for monoclonal antibodies (mAbs) and other therapeutic proteins (TPs) to improve patient compliance and reduce treatment costs [1]. One challenge in the study of mAbs is understanding, in mechanistic and quantitative terms, the factors that affect their bioavailability, which reflects the loss of a fraction of the injected drug before reaching the systemic circulation. There is no sufficiently reliable in vitro assay or preclinical species that is predictive of mAb bioavailability in humans. It is known that bioavailability correlates with the systemic clearance of a mAb [2], which is a measure of intrinsic stability, hydrophobicity, and any charge effect [3,4]; however, this relationship is empirical and might not be predictive for novel mAbs. The lack of mechanistic modeling of bioavailability was recently identified as one of the key knowledge gaps by the SC consortium [5].

Macrophages and other APCs of monocyte lineage have been demonstrated to be important for bioavailability in rodents [6,7]. They are mainly located in lymph nodes, including those draining the SC site; although, macrophages are also present in the dermis of the skin and can migrate to the injection site as a reaction to the mechanical pressure of the injection needle [8]. Macrophages contain the neonatal Fc receptor (FcRn), which protects immunoglobulin G (IgG) and other Fc-containing TPs from lysosomal degradation [6,7]. APCs endocytose large volumes of surrounding fluid [9] whilst scanning the environment for foreign antigens as part of the innate immune system [10,11].

Physiologically based pharmacokinetic (PBPK) models can be expected to predict the bioavailability of novel mAbs or altered bioavailability in disease populations once the respective mechanisms are formulated and incorporated into these models. However, at present, mechanistic a priori predictions of bioavailability are challenging [5], because only descriptive pre-systemic clearance pathways have been fitted to observed data and these are not predictive for novel mAbs. It has been found that lymphatic clearance is correlated to bioavailability rather than clearance at the injection site [12,13,14], which is in accordance with preclinical data [6,7].

Another challenge exists in defining the SC injection site volume in a PBPK model, because its dimensions are, to an extent, arbitrary, as there is no hard boundary between exposed and unexposed regions of the skin. An arbitrary value of 5 mL has often been used in the past [15], which assumes a three- to five-fold dilution of the typical SC dose of 1–1.5 mL. The volume of the injection site is determined by the distribution space between the site of injection and the blood vessels in case of peptides, small proteins, and oligonucleotides; or between the site of injection and the initial lymphatics in case of mAbs and other larger TPs [16]. The pre-systemic clearance could depend on the administration site because of different physiological characteristics such as lymph flow, the number of draining lymph nodes, or the number of macrophages in different parts of the skin [17].

The primary aim of this study was to develop a mechanistic PBPK model for estimating mAb bioavailability that includes the local lymphatic system with APCs in SC-injection-site-draining lymph nodes. In addition, the properties of four different injection sites (the arm, abdomen, back, and thigh) were considered to simulate site-specific differences in mAb pharmacokinetics and bioavailability.

## 2. Material and Methods

Four steps were taken in the construction of this SC PBPK model. First, a literature review was conducted to understand the physiological processes occurring following SC dosing. Second, the model was developed and parameterized with published physiological data. Third, a case study was conducted with IgG to test the model performance without optimization of any physiological or drug parameters. And fourth, we explored drug-related factors that have an impact on bioavailability.

### 2.1. Model Structure

**Figure 1:**
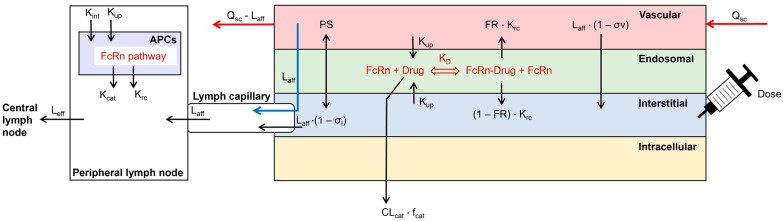
Structure of the subcutaneous absorption model, coupled to a whole-body PBPK model in the Simcyp Simulator V22. Arrows represent the disposition of the simulated TP. APCs = antigen-presenting cells, CL_cat_ = catabolic clearance, f_cat_ = fraction of catabolic clearance, FR = fraction recycled, K_cat_ = catabolic clearance, KD = binding affinity to FcRn at pH 6.0, K_rc_ = recycling rate, K_int_ = internalization rate, K_up_ = fluid-phase endocytosis uptake rate, L_aff_ = afferent lymph flow, L_eff_ = efferent lymph flow, PS = permeability surface area product of small and large pores, Q_sc_ = blood flow to the SC site, σ_l_ = lymphatic reflection coefficient, σ_v_ = vascular reflection coefficient through small and large pores.

The structure of a previously published SC injection site model was enhanced by the inclusion of local lymph capillaries and a peripheral draining lymph node compartment next to the injection site (Figure 1; [15]). APCs were incorporated as an additional compartment in the peripheral lymph node. The processes in APCs included fluid-phase endocytosis, FcRn recycling, and catabolism of unbound mAbs. Endogenous IgG was included during the simulation [18]. The SC site model was coupled to a whole-body PBPK model, implemented in the Simcyp Simulator V22 (Certara UK Ltd., Sheffield, UK). The SC site had a permeability-limited structure, similar to other compartments of the PBPK model. It contained the vascular space, the endothelial endosome, and the interstitial space. Dose administration was included in the interstitial space and a bolus injection was always simulated. The two-pore filtration–diffusion description was used to describe the distribution of dosed TPs between the vascular and interstitial spaces of the injection site [19]. The FcRn pathway was incorporated into the endothelial endosomal compartment [18]. The endothelial cell volume was parameterized as 5% of the vascular volume and endosomes covered 25% of the cell volume [20,21]. The ordinary differential equations to describe the disposition of the mAb at the SC site can be found in the Supplementary Materials.

### 2.2. Model Parameterization

First, the SC site volume was defined by using a study that investigated the travel distance of labeled albumin between the arm/back and sentinel lymph nodes in humans [22]. Four individuals (two women) aged 45–70 years had a mean (min–max) travel distance of 19.6 (11.5–32.5) cm and a mean (min–max) travel time of 2.0 (1.0–2.3) minutes after the injection of labeled albumin into the arm. Measurements in the back were conducted in six individuals (four women) aged 31–72 years. The mean (min–max) travel distance was 23.9 (11.5–40.0) cm, and the mean travel time (min–max) was 4.4 (1.0–10.5) minutes. The high variability observed in that study could not be correlated to age or sex. Given that the distance was measured to sentinel lymph nodes, these distance values will likely include the initial lymphatics. By assuming a circular distribution radius and by taking a subcutaneous thickness of 1.91 mm [23], the SC site volume could be estimated (Table 1). An average value of arm-and-back injection site volume was assumed for the abdomen and thigh in the absence of measured values for these two sites. The fraction of the injection site volume accounted for by the vascular, endosomal, and interstitial compartments was assumed to be the same as that accounted for by the skin.

Second, the blood flows were parameterized. Blood flows were defined as 9.2 mL/min/100 g, 2.9 mL/min/100 g, 7.4 mL/min/100 g, and 6.0 mL/min/100 g for the arm, abdomen, back, and thigh, respectively [23]. These values were incorporated as a % of cardiac output by using the derived SC site volume, a skin density of 1.1 g/mL [24], and the cardiac output of a population representative of the Simcyp Simulator V22 (397.5 L/h).

Third, the local lymphatic system was incorporated into the PBPK model. An important parameter is the number of peripheral lymph nodes that a mAb has to distribute through before reaching the central lymphatic system and consequently the blood circulation. This number of lymph nodes was found to be in the ranges of 25–36, 18–26, 20–29, and 18–26 for the arm, abdomen, back, and thigh, respectively [25]. The study that was used to measure the travel distance and time of labeled albumin to sentinel lymph nodes was used to determine the afferent lymph flow. The mean flow in the arm and back was 4.80 × 10^−5^ L/h and 4.87 × 10^−5^ L/h, respectively [22]. Each mean flow value was converted to the % of total lymph flow by multiplying it with the number of lymph nodes and using the value of total lymph flow from the Simcyp Simulator V22 (0.391 L/h). The average arm-and-back lymph flow was used for the abdomen and the thigh in the absence of measured values for these sites. The efferent lymph flow was estimated, using a mathematical model, to be 93% of the afferent lymph flow in mice [26]. The remaining 7% of the afferent lymph flow travels to the systemic circulation at the peripheral lymph nodes. Convection through aquaporins was incorporated to maintain fluid mass balance between afferent and efferent lymph flow in the model. Aquaporins have a radius of 0.2 nm [27], which means that mAbs are excluded from distribution. These calculations are similar to the two-pore hypothesis. The lymph capillary volume was parameterized by using the aforementioned study with labeled albumin, which reported a relationship between lymph flow and the lymph capillaries; the study measured the travel distance to sentinel lymph nodes and was thus corrected by the density of lymph vessels in the skin [28]. The average volume of a cervical lymph node is 0.284 mL, with high interindividual variability [29]. This average volume was multiplied with the number of lymph nodes to obtain the volume of the peripheral SC-site-draining lymph node compartment. The peripheral lymph nodes collect the fluid from interstitial-space watershed areas to filter it and APCs remove any foreign particles or proteins [30].

**Table 1:** Physiological input parameters for the four different SC sites.

Parameter	Arm	Abdomen	Back	Thigh	References
SC site volume (L/kg)	0.00077	0.00099	0.00115	0.00099	Calculated based on [22]
Vascular volume (% of the SC site)	5	5	5	5	[15]
Endosomal volume (% of the SC site)	0.06	0.06	0.06	0.06	[20,21]
Interstitial volume (% of the SC site)	59.5	59.5	59.5	59.5	[15]
Blood flow (% of the cardiac output)	0.088	0.035	0.105	0.073	[23]
Afferent lymph flow (% of the total lymph flow)	0.191	0.205	0.262	0.272	[22]
Efferent lymph flow (% of the afferent lymph flow)	93.0	93.0	93.0	93.0	[26]
Lymph capillary volume (L/kg)	0.000352	0.000392	0.000415	0.000387	[22]
Peripheral lymph node volume (L/kg)	0.000116	8.38 × 10^−5^	9.52 × 10^−5^	8.38 × 10^−5^	[25]

It is rather difficult to measure the number of macrophages in a lymph node. In mice, 42,000 subcapsular sinus macrophages have been measured in a single lymph node [31], which represent 29.7% of all macrophages [32]. The volume of a representative macrophage and lymph node in mice has been measured to be 1.66 × 10^−9^ [33] and 0.00095 mL [34], respectively. This leads to a macrophage volume of 24.5% of the peripheral lymph node volume in mice, and we assumed that the macrophage volume in humans would be similar to this value (Table S1). The fluid-phase endocytosis rate into macrophages has been reported to be 25% of their volume per hour [9]. Macrophages have been reported to contribute to site-specific bioavailability differences; however, we assumed the macrophage parameters to be similar between injection sites given the limited experimental data. Other cells present in lymph nodes also contribute to macropinocytosis and bioavailability, like dendritic cells and B-cells. In that sense, the “macrophage” population in the PBPK model includes all cells which are involved in high-rate macropinocytosis and mAb recycling.

### 2.3. Bioavailability Calculation

Bioavailability is defined as the fraction of a dosed drug that reaches the systemic circulation. We therefore split the concentration at the injection site into one coming from the dose and one returning from the systemic circulation. The concentration coming from the dose considered only outflows from the injection site and was used to calculate the bioavailability (see Supplementary Materials). The accumulated eliminated amount of the different clearance pathways was integrated, namely catabolic clearance at the SC site in macrophages and in the central lymph node compartment.

### 2.4. Model Validation

The model was evaluated using clinically observed data for subcutaneously dosed mAbs [35] as well as the clearance of labeled IgG from the SC injection site [15]. Visual predictive checking was used to analyze the model’s performance for the observed vs. predicted data. The published peak concentration (C_max_) and time to C_max_ (t_max_) were compared against the model’s predictions and predictions were considered successful if any differences they showed were within a 1.5-fold alteration of the observed data.

C_max_ and t_max_ for IgG were predicted at the four different SC sites and the results were compared against clinically observed data for 47 different IgG-based mAbs for which site-specific pharmacokinetic data had been published [17].

We evaluated the impact of the FcRn recycling pathway in the vascular endothelium at the SC site and in APCs by simulating four virtual patient cohorts along the lines of the preclinical study by Richter et al. [6], who studied in Tg32 transgenic mice the relative fractions of human IgG catabolized in bone-marrow-derived hematopoietic cells versus endothelial and parenchymal cells in the body. The relative changes in bioavailability were compared against the findings of Richter et al. [6].

### 2.5. Sensitivity Analyses

Pre-systemic first-pass catabolism in draining lymph nodes by resident APCs appears to be a critical determinant of the SC bioavailability of mAbs. Sensitivity analysis was performed on the pinocytotic uptake rate (range: 0–2.5 1/h) and on the binding affinity to FcRn (range: 0.128–2.0 μM). Additionally, sensitivity analysis was performed on the dose, which ranged from 0.01 to 1000 mg, to investigate the general trend, knowing that the used dose boundaries are not clinically relevant. All sensitivity analyses were performed using the arm as a representative SC injection site.

## 3. Results

Three steps were performed to evaluate the model.

**Figure 2:**
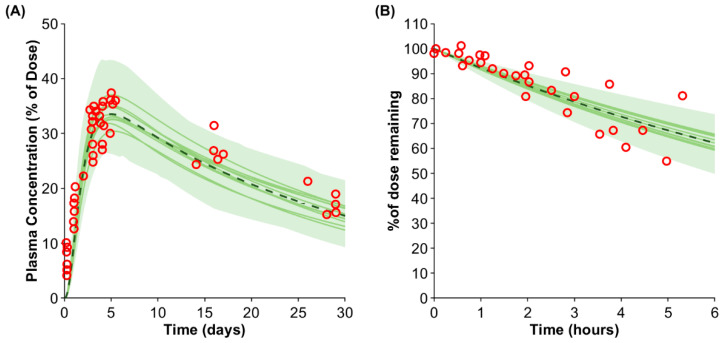
Predicted vs. observed IgG plasma concentration (**A**) and removal rate from the arm (**B**). The red markers represent the observed data [15,35]. The solid lines, the dashed line, and the shaded area show the mean of each virtual trial and the mean and the confidence interval of the entire virtual population.

First, the model performance was validated against the IgG plasma concentration after SC injection [35]. The predicted C_max_ and t_max_ of 33.5% of the injected dose (predicted/observed ratio: 0.96) and after 5.0 days (predicted/observed ratio: 0.98) agreed with the published clinical data (Figure 2A).

Second, the removal of IgG from the SC dosing site in the arm was simulated and compared against clinically observed data [15]. The predicted average removal rate agreed with the observed data; however, the variability was slightly underpredicted (Figure 2B).

Third, the predicted impact of site-specific administration was compared against clinically observed data from 47 mAbs ([17]; Table 2). The C_max_ ratio for the arm/abdomen and the thigh/abdomen agreed with the observed data (predicted/observed ratio: 0.99). The arm was predicted to have a higher t_max_ than the thigh in accordance with the observed data; however, the arm had the highest observed t_max_, but the model predicted the highest t_max_ in the abdomen. The trend in the AUC was captured by the model with a predicted AUC ratio for the arm/abdomen of 0.95 (predicted/observed ratio: 0.97) and a predicted AUC ratio for the thigh/abdomen of 1.06 (predicted/observed ratio: 0.94). Overall, the site-specific differences agreed with clinical data but were predicted to be marginal based on the physiological data of the respective site.

The impact of the APC compartment in the model was also investigated. The mouse study of Richter et al. was simulated in humans with subcutaneously dosed IgG to analyze whether the general trend in bioavailability observed in transgenic mice can be captured [6]. Richter et al. had four cohorts of mice: cohort 1 with wildtype mice, cohort 2 comprising mice with FcRn knocked-out in hematopoietic cells, cohort 3 comprising mice with FcRn knocked-out in endothelial cells, and cohort 4 comprising mice with a global FcRn knock-out. We simulated the bioavailability of IgG in humans using a similar study design to test the contribution of APCs to bioavailability. The predicted bioavailability in cohorts 1–4 was 65.1%, 25.6%, 66.1%, and 24.8%, respectively. Thus, cohort 1 and 3, as well as cohort 2 and 4, share roughly the same bioavailability, similar to the preclinical findings of Richter et al. The predicted/observed ratios for the bioavailability in cohorts 2, 3, and 4 relative to cohort 1 were 0.87, 1.00, and 0.60, respectively.

**Figure 3:**
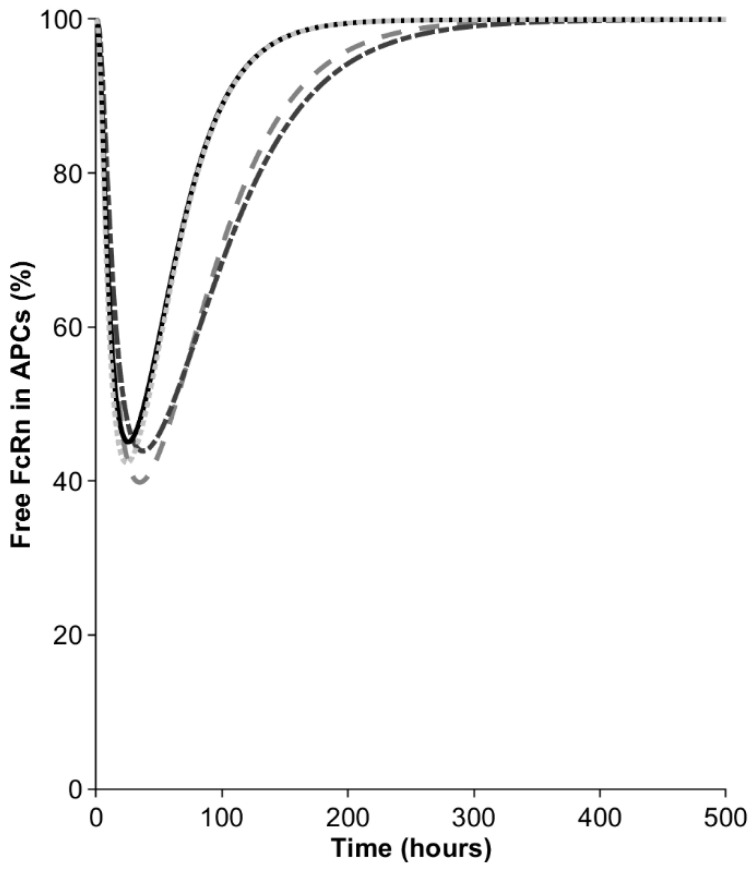
Percentage of free FcRn in antigen-presenting cells (APCs) in peripheral lymph nodes draining the SC site. Dash dotted line = arm, dashed line = abdomen, solid line = back, dotted line = thigh.

In the next step, we investigated the contribution of macrophages to bioavailability in the model. After the injection of 10 mg, the mAb concentration in the interstitial space of the SC site was around 1000 mg/L but became diluted when distributing into the lymph capillaries and peripheral lymph nodes, draining the SC site. Nevertheless, even the diluted mAb concentration in APCs (550–750 mg/L, depending on the SC site) remained sufficiently high to result in a transient saturation of the FcRn recycling pathway, as manifested by the decrease in the free fraction of the receptor (Figure 3). The free FcRn concentration was predicted to drop to 40–45%, depending on the injection site. The minimal free FcRn level was reached 35 h after the injections into the arm and abdomen and this was observed half a day earlier for the back and thigh. The saturation of FcRn in the macrophages was transient and the free FcRn was predicted to return to baseline after 8–12 days, depending on the SC site. The transient saturation of FcRn in the SC-dosing-site-draining lymph nodes results in elevated catabolism in macrophages and reduces the bioavailability to below 100%.

**Figure 4:**
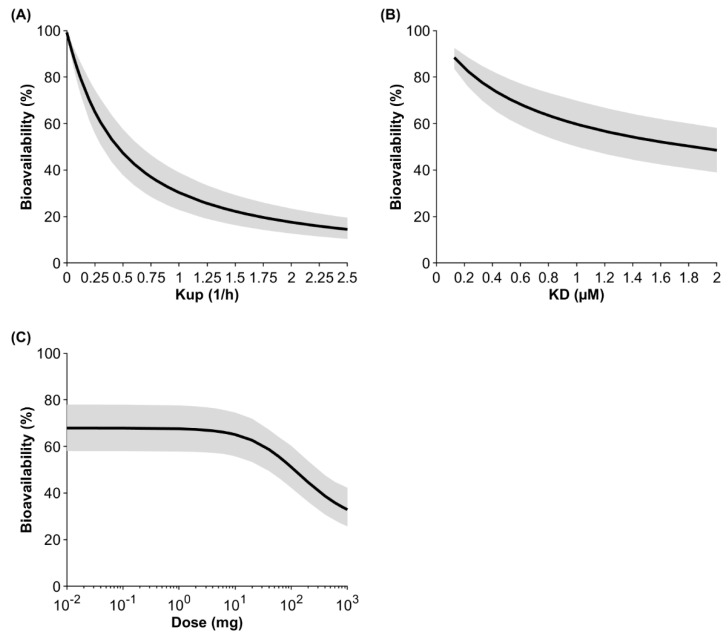
Population sensitivity analysis on the fluid-phase endocytosis uptake (Kup) into APCs (**A**), the binding affinity to FcRn (**B**), and the dose (**C**) after injection of IgG into the arm. The black line shows the mean and the gray shaded area denotes the 95% confidence interval of a virtual population containing 100 individuals (50% female) aged 20 to 50 years.

Sensitivity analyses were conducted investigating the impact of the endocytosis uptake into macrophages, the binding affinity to FcRn, and the administered dose on bioavailability (Figure 4). At a default uptake rate of 0.25 1/h, a binding affinity to FcRn of 0.728 μM, and a dose of 10 mg, the model predicted the bioavailability to be 65.1% for IgG injected into the arm. If the uptake rate was two-fold lower, the predicted bioavailability increased to 79.2%, and if the uptake rate was two-fold higher, the predicted bioavailability decreased to 47.3%. If the KD value for IgG (0.728 µM) was two-fold lower, the predicted bioavailability increased to 76.1%, and if the KD value was two-fold higher, bioavailability decreased to 53.6%. Dose had a marginal impact on bioavailability up to 2 mg. The predicted bioavailability decreased by 4.1% and 24.7% when the dose was increased to 10 mg and 100 mg, respectively. At a very high and theoretical dose of 1 g, bioavailability was predicted to be 32.9%.

## 4. Discussion

SC injection is the preferred route of administration for mAbs, but a priori predictions of bioavailability remain challenging. In this study, we evaluated and modeled the role of APCs in peripheral lymph nodes draining the SC site and demonstrated that a high drug concentration after injection transiently saturates the FcRn recycling pathway, resulting in a higher fraction of the endosomal mAb being degraded. This results in the bioavailability for mAbs being in the range of 60–70% of the dose given.

Predicting the bioavailability of novel mAbs from first principles is the next step for PBPK models, wherein the current models predominantly rely on fitted clearances and empirical relationships. Many different approaches have been taken to explore the impact of factors on bioavailability. Li et al. developed a whole-body PBPK model that included an SC site and fitted a degradation rate to represent catabolism at the injection site and in the local lymphatic system [36], while Varkhede et al. and Hu et al. implemented an SC absorption compartment that included the local lymphatic system [12,13]. In the latter approach, Varkhede et al. implemented lymphatic degradation and not clearance at the injection site as the main factor affecting bioavailability [12], with Hu et al. taking a similar approach by linking the empirically fitted lymphatic clearance [13]. In both cases, the lymphatic clearance—and thus, bioavailability—was linked to the charge as the sole predictor for a mAb.

While protein charge can play a role in mAb pharmacokinetics, we decided to focus on the role of cellular catabolism as exercised by APCs in the peripheral draining lymph nodes of the SC dosing site, because charge interaction measured by heparin binding can only be used empirically in PBPK models [37] and other factors such as hydrophobicity might also have an impact [4]. Lymphatic circulation forms an integral part of antibody circulation, with lymph nodes collecting the fluid from interstitial-space watershed areas before passing through the lymph nodes, where the resident APCs constantly sample the surrounding medium for any foreign particles or proteins as part of the early innate immune response [30]. Any mAb or protein with a great enough molecular weight to enter the lymphatic system [16] will therefore travel through the peripheral lymph nodes and be exposed to APCs before reaching the systemic circulation. APCs macropinocytose large volumes of the surrounding medium (20–40% of cell volume per hour) [9] and contain FcRn [38] to recycle endogenous compounds such as albumin and IgG, and will therefore also recycle any TP with an Fc domain. In our model, we track how a subcutaneously dosed mAb at a very high initial concentration travels from the dosing site to the draining lymph nodes, where the transient spike in total IgG causes a temporary fall in the free fraction of FcRn in APCs. As a result, a significant fraction of the endocytosed mAb cannot bind the receptor for recycling and is catabolized in lysosomes. This loss is irreversible and manifests itself as decreased bioavailability (i.e., a decreased fraction of the dose entering systemic circulation), which is typically around 60–70% for therapeutic mAbs. In comparison with the previous PBPK models that demonstrated lymphatic clearance to be important [12,13], we derived the degradation pathway a priori based on the macropinocytosis rate into APCs and saturable FcRn-mediated recycling.

The link to the protein charge found in previous models [12,13,39] can be explained by the dependency between the fluid-phase pinocytosis into APCs and the charge distribution of an antibody [40], but this effect is rather weak and correlates most strongly with the charge carried by the complementary determining regions in the antibody paratope region, which contributes modestly to overall protein charge. Nevertheless, it is possible that positively charged surface areas at the variable site of an antibody can interact with negatively charged components of cell membranes such as phospholipids or the glycocalyx. The latter can be evaluated by measuring mAb retention time on heparin affinity chromatography columns, which correlates with systemic clearance [3]. Previous PBPK models have linked the heparin retention time to the fluid-phase endocytosis uptake rate into endothelial cells [37,41]. The same effect can be expected for pinocytosis into APCs. The more positive the charge of an antibody is (or the higher the relative heparin retention time), the greater the uptake into APCs. This would result in a greater transient saturation of the FcRn pathway and lower the bioavailability (Figure 4). Antibodies with enhanced uptake into APCs in SC-site-draining lymph nodes may be predisposed to local uptake during the first pass and continued uptake during the elimination phase, establishing the correlation between bioavailability and terminal half-life. In the future, in vitro methods such as heparin column chromatography could be used as a measure of adhesion-related cellular uptake of proteins and more readily implemented in predictive PBPK models.

Another important factor that determines bioavailability in our model is the binding affinity to FcRn (Figure 4). A 10-fold-higher affinity for FcRn, with everything else being equal, results in an enhanced bioavailability of 92.5%. However, the “bottleneck” is the uptake into APCs, because only what distributes into the cell can bind to FcRn.

The current model has some limitations that should be acknowledged. First, APCs were only incorporated as a pre-systemic clearance pathway and are not included directly in our model for the homeostasis of IgG. Second, the physiological parameters used in this model carry a degree of uncertainty. The SC site volume in our model is based on the distribution space of a mAb between the site of injection and the initial lymphatics. As smaller proteins and peptides can diffuse through the pores of the vascular endothelium, resulting in a t_max_ within hours, the modeled distribution space, and thus the modeled SC site volume, is expected to be different compared with that of an actual mAb. We used data based on labeled albumin to parameterize the SC site volume, and thus this value is applicable for large proteins, including mAbs [16,22]. Data for smaller TPs were not found and thus the current value should be used with caution. In addition, the distribution of labeled albumin was measured until the peripheral lymph node and thus the volume can be expected to already include the initial lymphatics [22]. The afferent lymph flow was determined based on labeled albumin from the same study [22]. Despite the uncertainty, the removal of IgG from the arm of breast cancer patients could be predicted well using our model without optimizing any parameters (Figure 2B), which gives confidence to the volume and lymph flow values we used for the arm. However, when comparing the different sites, the highest t_max_ was predicted in the abdomen but was observed after injection into the arm. t_max_ would mainly be impacted by the afferent lymph flow. Another physiological parameter with uncertainty is the number of APCs in lymph nodes, which is difficult to measure and was obtained from previous measurements taken in mice. Ideally, the model would require observed concentration data of IgG in the lymph nodes draining the injection site, but as these data are currently not available, we rely on the assumptions made in the model and the observed plasma concentration time-course values, which were predicted in accordance with the observed data for an IgG-based mAb. One further limitation is that the model verification beside IgG focused on the C_max_ and t_max_ of 47 mAbs and not the concentration–time profiles of these mAbs. C_max_ and t_max_ values embed bioavailability, a key characteristic of subcutaneous absorption, and determining this was the goal of our model evaluation. Once in circulation, there is no further significant impact of the dosing site on the mAb plasma concentration time course, and thus C_max_ and t_max_ evaluation can be considered sufficient for the evaluation of subcutaneous absorption. A strength of the verification method we used is that plasma C_max_ and t_max_ values can be predicted without empirical fitting of any absorption-related parameters.

Given the limitations, this model describes a typical IgG-based mAb and is most useful for evaluating the impact of factors like dose volume, concentration, and interval time, in addition to generic parameters like the FcRn-binding affinity. We also envisage the possibility that if the excipients play a dominant role in keeping the mAb soluble in the formulation, these would be lost from the dosing site more rapidly than the mAb, thus increasing the risk of aggregation and dosage loss.

## 5. Conclusions

In conclusion, the incorporation of APCs in the SC-dosing-site-draining lymph nodes allows prediction of the bioavailability of IgG. The reason for the reduced bioavailability of mAbs is described as a consequence of the transient saturation of the FcRn receptor in APCs, which leads to a higher catabolism and consequently a lower bioavailability. Important antibody-related factors that have an impact on the bioavailability are the uptake rate into APCs and the binding affinity to FcRn. Higher doses, a positive charge distribution, or a low binding affinity to FcRn can result in a lower bioavailability. The presented model describes the bioavailability of a mAb mechanistically and represents a transformative advancement in drug discovery and development. It offers a valuable tool that can be used to optimize dosing regimens, which will ultimately lead to safe and efficacious therapies for patients.

## Figures and Tables

**Table 2:** Site-specific IgG predictions against observed data from 47 IgG-based antibodies [17].

	C_max_ Ratio		t_max_ Ratio		AUC Ratio	
	Observed	Predicted	Observed	Predicted	Observed	Predicted
Arm/Abdomen	0.92	0.96	1.26	0.98	0.98	0.95
Thigh/Abdomen	1.14	1.13	0.97	0.77	1.13	1.06

## Data Availability

Data can be made available upon request from the authors if not given in this publication.

## Supplementary Materials

The following supporting information can be downloaded at https://www.mdpi.com/article/10.3390/antib13030070/s1: Section S1. Ordinary differential equations for the proposed subcutaneous absorption model. Section S2. Bioavailability calculation. Section S3. List of physiological parameters used to parameterize the antigen-presenting cell compartment. Table S1: Physiological parameters to parameterize the antigen-presenting cell compartment. Reference [42] is cited in the Table S1.
