# Supplementary material for: A Physiologically Based Pharmacokinetic Model Relates the Subcutaneous Bioavailability of Monoclonal Antibodies to the Saturation of FcRn-Mediated Recycling in Injection-Site-Draining Lymph Nodes
Source: Antibodies (Basel). 2024 Aug 15;13(3):70. doi: 10.3390/antib13030070 (PMC11348173; doi:10.3390/antib13030070)
Supplement: Supplementary file 1 [file antibodies-13-00070-s001.zip › antibodies-3122563-supplementary.pdf]

Supplementary material

Article title: A Physiologically Based Pharmacokinetic Model Relates the Subcutaneous Bioavailability of Monoclonal Antibodies to the Saturation of FcRn-Mediated Recycling in Injection-Site-Draining Lymph Nodes

Authors: Felix Stader, Cong Liu, Abdallah Derbalah, Hiroshi Momiji, Xian Pan, Iain Gardner, Masoud Jamei, Armin Sepp

Affiliations: Certara UK Ltd, Simcyp Division
Level 2 Acero
1 Concourse Way
Sheffield, S1 2BJ
United Kingdom

Corresponding author: Felix Stader
Certara UK Ltd, Simcyp Division
Level 2 Acero
1 Concourse Way
Sheffield, S1 2BJ
United Kingdom
E-mail: felix.stader@certara.com
Phone: +44 (0)-114-460-0200

S1. Ordinary differential equations for the subcutaneous absorption model

The ordinary differential equations (ODEs) for the whole-body PBPK model were described elsewhere (1, 2). Here, we describe the ODEs for the subcutaneous (SC) absorption model.

S1.1. Interstitial space at the SC site

$$V_{IS} \cdot \frac{dC_{IS}}{dt} = Jc \cdot C_{VA} + Jd \cdot (C_{VA} - C_{IS}) - Ji \cdot C_{IS} - Kup_{EN} \cdot V_{IS} \cdot C_{IS} + (1 - FR) \cdot Krc \cdot V_{EN} \cdot fb_{EN} \cdot C_{EN}, \quad \text{(S1)}$$

V_{IS} = volume of the interstitial space (IS) of the injection site,

V_{EN} = volume of the endosomal space (EN) of the injection site,

C_{IS} = mAb concentration in the interstitial space (IS) of the injection site,

C_{VA} = mAb concentration in the vascular space (VA) of the injection site,

C_{EN} = mAb concentration in the endosomal space (EN) of the injection site,

Jc = protein distribution by convection (calculated according to the two-pore hypotheses),

Jd = protein distribution by diffusion (calculated according to the two-pore hypothesis)

Ji = protein distribution into the initial lymphatics ($Ji = (1 - \sigma_i) \cdot L_{aff}$ with σ_i=lymphatic reflection coefficient and L_{aff}=afferent lymph flow,

Kup_{EN} = fluid-phase endocytosis uptake into the endosomal space (EN),

FR = fraction recycled,

Krc = recycling rate,

fb_{EN} = fraction of mAb bound to the FcRn receptor in the endosomal space. Determined by Newton iteration (see Section S1.7),

Initial condition: $C_{IS}(t = 0) = \frac{Dose}{V_{IS}}$.

S1.2. Endosomal space at the SC site

$$V_{EN} \cdot \frac{dC_{EN}}{dt} = Kup_{EN} \cdot V_{VA} \cdot C_{VA} + Kup_{EN} \cdot V_{IS} \cdot C_{IS} - Krc \cdot V_{EN} \cdot fb_{EN} \cdot C_{EN} - CLcat \cdot fu_{EN} \cdot C_{EN}, \quad \text{(S2)}$$

V_{EN} = volume of the endosomal space (EN) of the injection site,

V_{VA} = volume of the vascular space (VA) of the injection site,

V_{IS} = volume of the interstitial space (IS) of the injection site,
C_{EN} = mAb concentration in the endosomal space (EN) of the injection site,
C_{VA} = mAb concentration in the vascular space (VA) of the injection site,
C_{IS} = mAb concentration in the interstitial space (IS) of the injection site,
Kup_{EN} = fluid-phase endocytosis uptake into the endosomal space (EN),
FR = fraction recycled,
Krc = recycling rate,
fb_{EN} = fraction of mAb bound to the FcRn receptor in the endosomal space. Determined by Newton iteration (see Section S1.7),
fu_{EN} = fraction of mAb not bound to the FcRn receptor in the endosomal space. Determined by Newton iteration (see Section S1.7),
CLcat = catabolic clearance,

Initial condition: $C_{EN}(t = 0) = 0$.

S1.3. Vascular space at the SC site

$$V_{VA} \cdot \frac{dC_{VA}}{dt} = Q \cdot C_{AB} - \left(Q - L_{aff}\right) \cdot C_{VA} - Jc \cdot C_{VA} - Jd \cdot (C_{VA} - C_{IS}) - Kup_{EN} \cdot V_{VA} \cdot C_{VA} + FR \cdot Krc \cdot V_{EN} \cdot fb_{EN} \cdot C_{EN}, \quad (S3)$$

V_{VA} = volume of the vascular space (VA) of the injection site,
V_{EN} = volume of the endosomal space (EN) of the injection site,
C_{VA} = mAb concentration in the vascular space (VA) of the injection site,
C_{AB} = mAb concentration in the arterial blood compartment (AB),
C_{IS} = mAb concentration in the interstitial space (IS) of the injection site,
C_{EN} = mAb concentration in the endosomal space (EN) of the injection site,
Q = blood flow,
L_{aff} = afferent lymph flow,
Jc = protein distribution by convection (calculated according to the two-pore hypotheses),
Jd = protein distribution by diffusion (calculated according to the two-pore hypothesis)
Kup_{EN} = fluid-phase endocytosis uptake into the endosomal space (EN),
FR = fraction recycled,
Krc = recycling rate,

fb_{EN} = fraction of mAb bound to the FcRn receptor in the endosomal space. Determined by Newton iteration (see Section S1.7),

Initial condition: $C_{VA}(t = 0) = 0$.

S1.4. Lymph capillary

$$V_{LC} \cdot \frac{dC_{LC}}{dt} = L_{aff} \cdot (1 - \sigma_l) \cdot C_{IS} - L_{aff} \cdot C_{LC}, \tag{S4}$$

V_{LC} = volume of lymph capillaries (LC),

C_{LC} = mAb concentration in the lymph capillaries (LC),

C_{IS} = mAb concentration in the interstitial space (IS) of the injection site,

L_{aff} = afferent lymph flow,

σ_l = lymphatic reflection coefficient,

Initial condition: $C_{LC}(t = 0) = 0$.

S1.5. Peripheral lymph node

$$V_{PN} \cdot \frac{dC_{PN}}{dt} = L_{aff} \cdot C_{LC} - L_{eff} \cdot C_{PN} - Kup_{AP} \cdot V_{PN} \cdot C_{PN} + Krc \cdot V_{AP} \cdot fb_{AP} \cdot C_{AP}, \tag{S5}$$

V_{PN} = volume of peripheral lymphnodes (PN),

V_{AP} = volume of antigen-presenting cells (AP),

C_{LC} = mAb concentration in the lymph capillaries (LC),

C_{PN} = mAb concentration in the peripheral lymphnodes (PN),

C_{AP} = mAb concentration in the antigen-presenting cells (AP),

L_{aff} = afferent lymph flow,

L_{eff} = efferent lymph flow,

Kup_{AP} = fluid-phase endocytosis uptake into antigen-presenting cells (AP),

Krc = recycling rate,

fb_{AP} = fraction of mAb bound to the FcRn receptor in antigen-presenting cells. Determined by Newton iteration (see Section S1.7),

Initial condition: $C_{PN}(t = 0) = 0$.

S1.6. Antigen-presenting cells compartment

$$V_{AP} \cdot \frac{dC_{AP}}{dt} = Kup_{AP} \cdot V_{PN} \cdot C_{PN} - Krc \cdot V_{AP} \cdot fb_{AP} \cdot C_{AP} - Kdeg_{AP} \cdot V_{AP} \cdot fu_{AP} \cdot C_{AP}, \tag{S6}$$

V_{PN} = volume of peripheral lymphnodes (PN),

V_{AP} = volume of antigen-presenting cells (AP),

C_{PN} = mAb concentration in the peripheral lymphnodes (PN),

C_{AP} = mAb concentration in the antigen-presenting cells (AP),

Kup_{AP} = fluid-phase endocytosis uptake into antigen-presenting cells (AP),

Krc = recycling rate,

$Kdeg_{AP}$ = degradation rate of the mAb in antigen-presenting cells,

fb_{AP} = fraction of mAb bound to the FcRn receptor in antigen-presenting cells. Determined by Newton iteration (see Section S1.7),

fu_{AP} = unbound fraction of the mAb (regarding FcRn binding) in antigen-presenting cells. Determined by Newton iteration (see Section S1.7),

Initial condition: $C_{AP}(t = 0) = 0$.

S1.7. Determine the FcRn binding by Newton iteration

Endogenous IgG and monoclonal antibodies (mAb) competitively bind to FcRn in the endosomal space and in antigen-presenting cells (APCs). We assume an equilibrium for this binding event to determine the unbound and bound fraction of IgG and mAb to FcRn. The fractions depend on the tissue concentration and thus, change with each time step and need to be solved at each time step.

We assume that one IgG/mAb molecule can bind to one FcRn molecule. The binding reactions can be written as:

$$R + G \overset{KD_G}{\longleftrightarrow} RG, \tag{S7}$$

$$R + A \overset{KD_A}{\longleftrightarrow} RA, \tag{S8}$$

where R = FcRn, G = IgG, A = mAb, RG = FcRn-IgG complex, RA = FcRn-mAb complex, and KD is the equilibrium dissociation constant, defined as:

$$KD_G = \frac{R \cdot G}{RG}, \tag{S9}$$

$$KD_A = \frac{R \cdot A}{RA}. \tag{S10}$$

The fraction bound (fb) is defined for IgG and mAb:

$$fb_G = \frac{RG}{G_T}, \quad \text{(S11)}$$

$$fb_A = \frac{RA}{A_T}, \quad \text{(S12)}$$

Where total IgG (G_T) and total mAb (A_T) are defined as followed:

$$G_T = G + RG, \quad \text{(S13)}$$

$$A_T = A + RA. \quad \text{(S14)}$$

We can now the free fraction of endogenous IgG and exogenous mAb:

$$fu_G = \frac{G}{G_T} = 1 - fb_G, \quad \text{(S15)}$$

$$fu_A = \frac{A}{A_T} = 1 - fb_A. \quad \text{(S16)}$$

Now, we express the free fraction of FcRn (x) by the fraction bound (fb):

$$x = \frac{R}{R_T} = \frac{KD_G \cdot RG}{R_T \cdot G} = \frac{KD_G}{R_T} \cdot \frac{fb_G}{1 - fb_G} = \alpha_G \cdot \frac{fb_G}{1 - fb_G}, \quad \text{(S17)}$$

$$x = \frac{R}{R_T} = \frac{KD_A \cdot RA}{R_T \cdot A} = \frac{KD_A}{R_T} \cdot \frac{fb_A}{1 - fb_A} = \alpha_A \cdot \frac{fb_A}{1 - fb_A}. \quad \text{(S18)}$$

The equation can be rearranged to express fb by x.

$$fb_G = \frac{x}{\alpha_G + x}, \quad \text{(S19)}$$

$$fb_A = \frac{x}{\alpha_A + x}. \quad \text{(S20)}$$

The total FcRn can be expressed as:

$$R_T = R + RG + RA, \quad \text{(S21)}$$

$$1 = x + \frac{G_T}{R_T} \cdot fb_G + \frac{A_T}{R_T} \cdot fb_A, \quad \text{(S22)}$$

with

$$\beta_G = \frac{G_T}{R_T}, \quad \text{(S23)}$$

$$\beta_A = \frac{A_T}{R_T}. \quad \text{(S24)}$$

To facilitate numerical solving Equation (S22) by Newton iteration, the following function is defined:

$$F(x) = x - 1 + \frac{\beta_G \cdot x}{\alpha_G + x} + \frac{\beta_A \cdot x}{\alpha_A + x}. \quad \text{(S25)}$$

The derivative of Equation (S23) is:

$$F'(x) = 1 + \frac{\beta_G \cdot \alpha_G}{(\alpha_G + x)^2} + \frac{\beta_A \cdot \alpha_A}{(\alpha_A + x)^2}. \quad \text{(S26)}$$

Then the Newton iteration method is applied based on the following recursion relation:

$$x^{(k+1)} = x^{(k)} - \left[F'\left(x^{(k)}\right)\right]^{-1} \cdot F\left(x^{(k)}\right), k = 0,1,2, \quad \text{(S27)}$$

and when the iteration converges:

$$x^{(k)} \to x, k \to \infty. \quad \text{(S28)}$$

S2. Bioavailability calculation

The concentrations of the injection site are split into a concentration that comes from the dose ($\hat{C}$) and a concentration that returns the drug from the systemic circulation ($\bar{C}$) to calculate bioavailability.

$$C_{XX} = \hat{C}_{XX} + \bar{C}_{XX}, \tag{S29}$$

Where XX represents any compartment of the injection site.

We take only the concentration, coming from the dose, to calculate bioavailability. Equations (S1)-(S6) are thus rewritten:

$$V_{IS} \cdot \frac{d\hat{C}_{IS}}{dt} = Jc \cdot \hat{C}_{VA} + Jd \cdot \left(\hat{C}_{VA} - \hat{C}_{IS}\right) - Ji \cdot \hat{C}_{IS} - Kup_{EN} \cdot V_{IS} \cdot \hat{C}_{IS} + (1 - FR) \cdot Krc \cdot V_{EN} \cdot fb_{EN} \cdot \hat{C}_{EN}, \tag{S30}$$

$$V_{EN} \cdot \frac{d\hat{C}_{EN}}{dt} = Kup_{EN} \cdot V_{VA} \cdot \hat{C}_{VA} + Kup_{EN} \cdot V_{IS} \cdot \hat{C}_{IS} - Krc \cdot V_{EN} \cdot fb_{EN} \cdot \hat{C}_{EN} - CLcat \cdot fu_{EN} \cdot \hat{C}_{EN}, \tag{S31}$$

$$V_{VA} \cdot \frac{d\hat{C}_{VA}}{dt} = -\left(Q - L_{aff}\right) \cdot \hat{C}_{VA} - Jc \cdot \hat{C}_{VA} - Jd \cdot \left(\hat{C}_{VA} - \hat{C}_{IS}\right) - Kup_{EN} \cdot V_{VA} \cdot \hat{C}_{VA} + FR \cdot Krc \cdot V_{EN} \cdot fb_{EN} \cdot \hat{C}_{EN}, \tag{S32}$$

$$V_{LC} \cdot \frac{d\hat{C}_{LC}}{dt} = L_{aff} \cdot (1 - \sigma_l) \cdot \hat{C}_{IS} - L_{aff} \cdot \hat{C}_{LC}, \tag{S33}$$

$$V_{PN} \cdot \frac{d\hat{C}_{PN}}{dt} = L_{aff} \cdot \hat{C}_{LC} - L_{eff} \cdot \hat{C}_{PN} - Kup_{AP} \cdot V_{PN} \cdot \hat{C}_{PN} + Krc \cdot V_{AP} \cdot fb_{AP} \cdot \hat{C}_{AP}, \tag{S34}$$

$$V_{AP} \cdot \frac{d\hat{C}_{AP}}{dt} = Kup_{AP} \cdot V_{PN} \cdot \hat{C}_{PN} - Krc \cdot V_{AP} \cdot fb_{AP} \cdot \hat{C}_{AP} - Kdeg_{AP} \cdot V_{AP} \cdot fu_{AP} \cdot \hat{C}_{AP}. \tag{S35}$$

The cleared amount is calculated for catabolism in the endosomal space of the injection site and in antigen-presenting cells.

$$R_{EN} = \int_{t=0}^{\infty} CLcat \cdot fu_{EN} \cdot \hat{C}_{EN}(t), \tag{S36}$$

$$R_{AP} = \int_{t=0}^{\infty} Kdeg_{AP} \cdot V_{AP} \cdot fu_{AP} \cdot \hat{C}_{AP}(t). \tag{S37}$$

The apparent bioavailability is now calculated as:

$$F = \left(1 - \left(\frac{1}{Dose}\right) \cdot (R_{EN} + R_{AP})\right) \cdot 100\%. \tag{S38}$$

S3. List of physiological parameters used to parameterize the antigen-presenting cell compartment

Table S1: Physiological parameters to parameterize the antigen-presenting cell compartment.

Parameter	Value	Reference
Volume (% of peripheral lymph node volume)	24.5	Calculated as described in the main text
Endosomal volume (% of macrophage volume)	25.0	(3)
FcRn (pmol / g tissue)	91.7	(4)
Endocytosis uptake rate (1/h)	0.25	(5)
Degradation rate (1/h)	0.2916	(6)

S4. References

(1) Li, L., Gardner, I., Dostalek, M. & Jamei, M. Simulation of monoclonal antibody pharmacokinetics in humans using a minimal physiologically based model. *The AAPS Journal* **16**, 1097-109 (2014).

(2) Gill, K.L., Gardner, I., Li, L. & Jamei, M. A bottom-up whole-body physiologically based pharmacokinetic model to mechanistically predict tissue distribution and the rate of subcutaneous absorption of therapeutic proteins. *The AAPS Journal* **18**, 156-70 (2016).

(3) Herring, N. & Paterson, D.J. *Levick's introduction to cardiovascular physiology* (CRC Press: 2018).

(4) Fan, Y.-Y., Farrokhi, V., Caiazzo, T., Wang, M., O'Hara, D.M. & Neubert, H. Human FcRn tissue expression profile and half-life in PBMCs. *Biomolecules* **9**, 373 (2019).

(5) Steinman, R.M., Brodie, S.E. & Cohn, Z.A. Membrane flow during pinocytosis. A stereologic analysis. *The Journal of Cell Biology* **68**, 665-87 (1976).

(6) Mellman, I.S., Plutner, H., Steinman, R.M., Unkeless, J.C. & Cohn, Z.A. Internalization and degradation of macrophage Fc receptors during receptor-mediated phagocytosis. *The Journal of Cell Biology* **96**, 887-95 (1983).
